# Inspection of Liner Wall Thinning and Interface Debonding in Bimetallic Lined Pipes Using Pulsed Eddy Current Testing

**DOI:** 10.3390/ma17112652

**Published:** 2024-05-30

**Authors:** Weifan Chen, Xiaofeng Zhou, Baixi Liu, Zhiping Li, Zan Luo, Zhiyuan Xu

**Affiliations:** 1 School of Mechanical Engineering and Mechanics, Xiangtan University, Xiangtan 411105, China; chenweifan95@163.com (W.C.);; 2 Xiangtan Iron and Steel Co., Ltd. of Hunan Valin, Xiangtan 411101, China; 3 Zhejiang University NGICS Platform, College of Control Science and Engineering, Zhejiang University, Hangzhou 310015, China; 4 Key Laboratory of Nondestructive Testing of Ministry of Education, Nanchang Hangkong University, Nanchang 330063, China

**Keywords:** bimetallic composite pipes, pulsed eddy current, corrosion, liner wall thinning, interface debonding

## Abstract

Bimetallic lined pipe (BLP) has been increasingly used in offshore and subsea oil and gas structures, but how to identify the invisible inner defects such as liner wall thinning and interface debonding is a challenge for future development. A nondestructive testing (NDT) method based on pulsed eddy current testing (PECT) has been proposed to face these difficulties. The inspection of the BLP specimen (AISI1020 base tube and SS304 liner) is implemented from outside of the pipe by using a transmitter–receiver-type PECT probe consisting of two induction coils. By simplifying the BLP specimen to stratified conductive plates, the electromagnetic field interaction between the PECT probe and specimen is analytically modeled, and the probe inspection signals due to liner wall thinning and interface debonding are calculated. In order to highlight the weak response (in microvolts) from the liner, the inspection signals are subtracted by the signal, which is calculated in the case of only having a base tube, yielding differential PECT signals. The peak voltage of the differential signal is selected to characterize the liner wall thinning and interface debonding due to its distinguishable and linear variation. Experiment verification is also carried out on a double-walled specimen simulated by a combination of a Q235 casing pipe and SS304 tubes of different sizes. The experimental results basically agree with the analytical predictions. The peak value of the PECT signal has an ascending and descending variation with the increase in the remaining liner wall thickness and debonding gap, respectively, while the negative peak value shows opposite changes. The peak value exhibits a larger sensitivity than the negative peak value. The proposed method shows potential promise in practical applications for the evaluation of the inner defects in BLP lines.

## 1. Introduction

The massive pipelines used in oil and gas (O&G) production and transportation are exposed to some of the most aggressive industrial environments. As is often the case in the subsea and offshore O&G industry, the inside is exposed to the corrosive ingredients of hydrocarbons, while the outside is required to withstand mechanical loads and impacts [1,2]. In order to simultaneously solve the corrosion problem and provide the required strength to maintain structural integrity, the use of a solid corrosion-resistant alloy (CRA) and bimetallic composite pipes is in high demand [3,4,5,6]. Bimetallic composite pipes, including a carbon steel (CS) base tube and an inner CRA layer, are considerably more economical than equivalent solid CRA pipes [5,6].

A double-walled, bimetallic composite pipe typically consists of a thin internal CRA pipe, with a typical thickness of 3 to 4 mm and a thick outer CS pipe. It can be manufactured through either mechanical or metallurgical bonding process, forming two main categories of CRA pipes—mechanically lined pipes and hot-roll cladded pipes, respectively [6,7,8]. The bimetallic lined pipe (BLP) is preferred in various applications because of its lower production costs, high productive efficiency, and availability in all sizes. Generally, BLP pipes provide excellent corrosion protection. However, due to manufacturing-related anomalies, wrong handling during storage, transportation and installation, and unsuitable selection of CRA material for the actual operational conditions, corrosion damage and geometric deformations can occur at the outer layer, internal layer and also the interface [1,2,9,10]. Among these failures, liner wall thinning and interface debonding, which are invisible and inaccessible from outside, are difficult to identify and, thus, pose great risk to the operational safety of BLP lines. Hence, test and inspection measures are required to ensure the structural integrity of BLP products. For the constructed BLP lines, an online inspection tool utilizing nondestructive testing (NDT) technologies is preferred in practice considering its high efficiency and cost effectiveness.

Ultrasonic testing (UT) can be used to inspect both the CRA layer and the CS base in clad pipes but will encounter inapplicability in lined pipes because the ultrasonic waves cannot negotiate the tiny gapped interface between the CS and the CRA [11]. Tian et al. [12] employed ultrasonic longitudinal interface waves for interlaminar crack detection in a steel–titanium pipe, in which the interface was tightly bonded through a mechanical process. This method is feasible and effective for inspecting interlaminar cracks but requires a free pipe spool without the outer layer to mount the surface wave transducers since, essentially, it belongs to the guided wave technique. The magnetic flux leakage (MFL) method is suited to the inspection of CS but does not work for the inspection of the CRA liner because the CRA is non-ferromagnetic. Radiographic inspection can provide intuitive images and profiles of the tested BLP but is restricted in field applications due to the risk of radiation exposure and a slow process [13]. Eddy current testing (ECT) is sensitive to all conductive metals but has limited penetration depth due to the skin effect and, thus, only responds to the near surface when applied to BLP lines. Keuter [14] presented a combined MFL/ECT pigging tool, which was capable of inspecting the CS and the CRA simultaneously. Nevertheless, the outer-diameter defect of the CRA liner still cannot be inspected. A likely solution to this challenge is using low-frequency ECT to obtain a larger penetration depth. Unfortunately, low frequency also causes other practical problems including low signal amplitude [15]. Compared with traditional ECT, pulsed eddy current testing (PECT) with pulsed- or square-wave excitation is found to have better performance in the inspection of deeply embedded defects, and its relevant applications have been frequently reported, especially on layered conductive structures [16,17,18,19,20]. Lebrun et al. [16] applied PECT to detect cracks in riveted aeronautical structures and found that small cracks (1 mm of radial extent) buried 5 mm (0.2 in.) in depth were detected. Yang et al. [17] developed a PECT-based giant magnetoresistive system for the automatic detection of small cracks under fasteners in multilayered structures at a depth of up to 10 mm. Xu et al. [18,19] employed PECT to detect corrosion in thermally insulated pipes without removing the wrapped weatherproofing jacket and insulation material.

Another issue that needs to be considered in practical applications is to inspect the O&G pipes from inside or outside. When the internal probe equipped with a pigging tool is placed inside the pipe, it will likely become stuck because a piping system has valves, joints, elbows, intake, and outlet structures, which are varied in inner diameter. Also, pressure differentials and flow can be too low to push a pigging tool through the pipe [21]. In this situation, an online NDT technique, which can continuously examine the pipeline from the outside, becomes a preferable choice.

The BLP consists of two conductive layers and, thus, indicates the use of PECT for inspecting defects embedded at every depth of the pipe wall. In this study, the PECT technique is used as an online method for inspecting BLP lines from the outside. The study specifically focused on the inspection of liner wall thinning and interface debonding. Analytical modeling was conducted to predict the PECT signal and correlate signal features with the residual liner thickness and the debonding gap. Experimental verification was also carried out to prove the modeling and validate the effectiveness of the presented method.

## 2. Analytical Modeling

**Figure 1:**
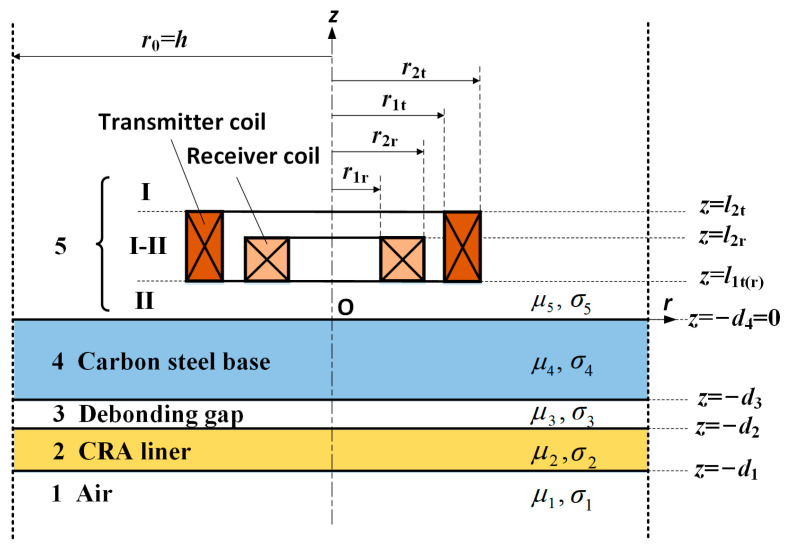
A TR probe above a four-layer structure.

Analytical modeling facilitates fast prediction of the inspection results ahead of experiments and helps understand the underlying physical mechanism, but it often requires simplifications or assumptions made on the geometry and boundary of the practical model. For ECT configured with a surface probe above a pipe, the pipe wall can be considered as a plate to simplify the problem, provided the pipe radius is far greater than the probe coil size [18,22,23]. In this case, the BLP can be approximated by a three-layer plate, with the interlayer representing the potential interface gap. A cylindrical coordinate system is applied with its z-axis perpendicular to the layered plate and the origin at the top surface, as shown in Figure 1. Taking the air into account, the media from bottom to top are the inner air, the CRA liner, the debonding gap, the CS base, and the outside air, respectively. Considering the low-frequency regime of ECT and to facilitate the modeling, the nonlinear and inhomogeneous attributions of the electromagnetic properties of the CS are neglected. Hence, all these media have constant magnetic permeabilities and electrical conductivities, and the gap layer is considered to be of the same properties as the air region.

A coaxial transmitter–receiver (TR) probe is positioned above the plate and divides the outside air into three sub-regions. The transmitter coil is driven by a periodic square-wave current, which is equivalent to the summation of Fourier series in terms of harmonics. For each harmonic component, the eddy current problem can be solved by using the classical Dodd and Deeds models [24]. Due to the coaxial configuration, the total induced voltage in the receiver coil contains not only the voltage change produced by the eddy currents induced in the plate below but also the induced voltage caused by the drive current in the transmitter coil. The latter part, usually called the null signal, is equal to the voltage in the receiver coil when the conductive object is absent or far away from the probe. It has a larger amplitude but does not carry the plate information. Hence, by subtracting the null signal, the desired eddy-current-induced voltage in the receiver coil is obtained [18]

(1) $$\begin{array}{l} \Delta U\left(\omega \right)\\ =\frac{j\pi \omega N_{t}N_{r}\mu_{0}I\left(\omega \right)}{\left(r_{2t}-r_{1t}\right)\left(l_{2t}-l_{1t}\right)\left(r_{2r}-r_{1r}\right)\left(l_{2r}-l_{1r}\right)}\\ \times \int_{0}^{\infty }\chi \left(\alpha r_{1t},\alpha r_{2t}\right)\chi \left(\alpha r_{1r},\alpha r_{2r}\right)\frac{\left(e^{-\alpha l_{2t}}-e^{-\alpha l_{1t}}\right)\left(e^{-\alpha l_{2r}}-e^{-\alpha l_{1r}}\right)}{\alpha^{6}}\Gamma \left(\alpha \right)d\alpha \end{array}$$

where $\chi \left(x_{1},x_{2}\right)=\int_{x_{1}}^{x_{2}}xJ_{1}\left(x\right)dx$ with $J_{1}\left(x\right)$ represent the first-order Bessel function of the first kind, $j=\sqrt{-1},$ ω denotes the angular frequency, $I\left(\omega \right)$ is the amplitude of the drive current for the component ω, $\mu_{0}$ is the permeability constant with a value of $4\pi \times 10^{-7}$ H/m, N refers to the number of turns of wire in the probe coils, and the subscripts t and r label the transmitter and receiver coil, respectively. $\Gamma \left(\alpha \right)$ is the reflection coefficient of the four-layer medium under the probe, which can be expressed as [18,25]

(2) $$\Gamma \left(\alpha \right)={\left.\frac{V_{12}\left(n,1\right)}{V_{22}\left(n,1\right)}\right|}_{n=5}=\frac{t_{11}\left(n,n-1\right)V_{12}\left(n-1,1\right)+t_{12}\left(n,n-1\right)V_{22}\left(n-1,1\right)}{t_{21}\left(n,n-1\right)V_{12}\left(n-1,1\right)+t_{22}\left(n,n-1\right)V_{22}\left(n-1,1\right)}$$

where

(3) $$\left\{\begin{array}{c} t_{i,k}\left(n,n-1\right)=\left[1+{\left(-1\right)}^{i+k}\beta_{n-1}\mu_{n}/\beta_{n}\mu_{n-1}\right]e^{{\left(-1\right)}^{k}\beta_{n-1}\left(d_{n-2}-d_{n-1}\right)},\;\;\;n=3,4,5\\ \;V_{i,2}\left(2,1\right)=\left[1+{\left(-1\right)}^{i+2}\beta_{1}\mu_{2}/\beta_{2}\mu_{1}\right] \end{array}\right.$$

with i and k are either 1 or 2, and $\beta_{n}=\sqrt{\alpha^{2}+j\omega \mu_{n}\sigma_{n}}$.

Due to the infinite boundary, Equation (1) comes as a Fourier–Bessel integral-form solution, which leaves a time-consuming task for subsequent integral computation. This challenge can be addressed by using the truncated region eigenfunction expansion (TREE) method [26]. Upon imposing a homogeneous Dirichlet condition for the magnetic field at $r_{0}=h$, Equation (1) can be replaced by a series expansion, as follows:

(4) $$\begin{array}{l} \Delta U\left(\omega \right)\\ =\frac{j2\pi \omega \mu_{0}N_{t}N_{r}I\left(\omega \right)}{\left(r_{2t}-r_{1t}\right)\left(l_{2t}-l_{1t}\right)\left(r_{2r}-r_{1r}\right)\left(l_{2r}-l_{1r}\right)}\\ \times \overset{\infty }{\underset{i=1}{\sum }}\chi \left(\alpha_{i}r_{1t},\alpha_{i}r_{2t}\right)\chi \left(\alpha_{i}r_{1r},\alpha_{i}r_{2r}\right)\frac{\left(e^{-\alpha_{i}l_{2t}}-e^{-\alpha_{i}l_{1t}}\right)\left(e^{-\alpha_{i}l_{2r}}-e^{-\alpha_{i}l_{1r}}\right)}{{\left[\left(\alpha_{i}h\right)J_{0}\left(\alpha_{i}h\right)\right]}^{2}\alpha_{i}^{5}}\Gamma \left(\alpha_{i}\right) \end{array}$$

where $\alpha_{i}$ is the *i*-th positive root of Bessel function $J_{1}\left(\alpha_{i}h\right)$, viz.

(5) $$J_{1}\left(\alpha_{i}h\right)=J_{1}\left(x_{i}\right)=0;\;\;\;\;\;\;\alpha_{i}=x_{i}/h$$

As the specimen material involved in modeling is presumed to be linear, the PECT system can be regarded as a linear system. According to the principle of superposition, the PECT voltage can be obtained by adding up all the voltages under different harmonic excitations, which forms a discrete voltage sequence in the frequency domain.

(6) $$\Delta U\left[\omega_{m}\right]=\left\{\Delta U\left(\omega_{1}\right),\;\Delta U\left(\omega_{2}\right),\;\ldots ,\;\Delta U\left(\omega_{M}\right)\right\}$$

where *M* is the number of truncated harmonic components and the base frequency $\omega_{1}=2\pi f$, with *f* representing the repetition frequency of the square-wave excitation.

Further, as the PECT signal is usually described in the time domain, by performing the inverse discrete Fourier transform (IDFT), the time evolution voltage is yielded

(7) $$\Delta U\left[t_{s}\right]=\frac{1}{M}\overset{M}{\underset{m=1}{\sum }}e^{j\frac{2\pi }{N}\left(s-1\right)\left(m-1\right)}\Delta U\left[\omega_{m}\right],\;s=1,2,\ldots ,M$$

The above formulae are implemented in MATLAB, where the DFT and IDFT are computed using the built-in functions FFT and IFFT, respectively. It should be noted that the omitted DC components, viz. when ω=0, need to be added during the implementation of FFT and IFFT. In addition, to smooth the Gibbs phenomenon caused by the truncation of the frequency spectrum, it is recommended to multiply the $I\left(\omega_{m}\right)$ by a decreasing factor $\gamma_{m}=\sin c\left(m\pi /M\right)$ [27].

## 3. Signal Characteristics

In this section, the established analytical model is used as a simulation tool to predict the inspection signal when the liner wall thinning or interface debonding occurs and, further, to select the signal waveform parameters as the potential features for evaluating and classifying the two types of failures. During simulations, the CS base and the CRA liner are specified as low-carbon AISI 1020 and SS304, respectively, whose material properties are given in Table 1. The AISI 1020 steel, which is commonly used in seamless pipes, shows insignificant magnetic variations in electromagnetic testing and is, therefore, reasonably considered, having a constant relative magnetic permeability [28]. The base plate is 10 mm thick, while the liner has a full thickness of 4 mm. In different simulation groups, the liner thickness and the interlayer gap are, respectively, altered to simulate the wall thinning and the interface debonding.

The probe coil parameters are listed in Table 2. When using ECT for thickness measurements, an appropriate excitation frequency must be selected, such that the standard depth of penetration of the eddy currents covers the entire thickness of the specimen [29]. The depth at which eddy current density has decreased to 1/e of the surface density is called the standard depth of penetration δ. It is defined as

(8) $$\delta =\frac{1}{\sqrt{\pi \mu \sigma f}}$$

where *f* refers to the excitation frequency, which is equal to ω/2π.

In the present case, the eddy currents first penetrate the AISI 1020 base and then go into the SS304 liner. Suppose the induced eddy current density at the surface of the CS base is equal to $J_{0}$; then, for current density below the surface

(9) $$J_{z}=J_{0}e^{-z/\delta_{1}}$$

where $\delta_{1}=1/\sqrt{\pi \mu_{4}\sigma_{4}f}$ with $\mu_{4}$ and $\sigma_{4}$ refer to the permeability and the conductivity of the CS base, respectively.

At the depth of the interface, $J_{z}$ decays to $J_{0}e^{-d_{3}/\delta_{1}}$ with *d*_3_ denoting the thickness of the CS base. Ignoring the backward reflection of eddy currents at the interface, the eddy current penetrated in the liner will have a density of $J_{0}e^{-d_{3}/\delta_{1}}e^{-z/\delta_{2}}$, where $\delta_{2}=1/\sqrt{\pi \mu_{2}\sigma_{2}f}$. Then, to ensure a complete eddy current penetration, the current density at the bottom of the liner should satisfy the following relation

(10) $$J_{0}e^{-d_{3}/\delta_{1}}e^{-\left(d_{1}-d_{2}\right)/\delta_{2}}\geq J_{0}/e$$

where $\left(d_{1}-d_{2}\right)$ denotes the liner thickness, as shown in Figure 1. Substituting $d_{3}=0.01$ m, $\left(d_{1}-d_{2}\right)=0.004$ m and the properties listed in Table 1 yields f≤2.11 Hz. Consequently, the maximum repetition frequency of the drive square-wave current is determined to be 2 Hz.

The amplitude and duty ratios of the excitation current are set to 3 A and 0.5, respectively, while the model parameters used for calculation are *h* = 40r_2t_ and *M* = 200.

**Figure 2:**
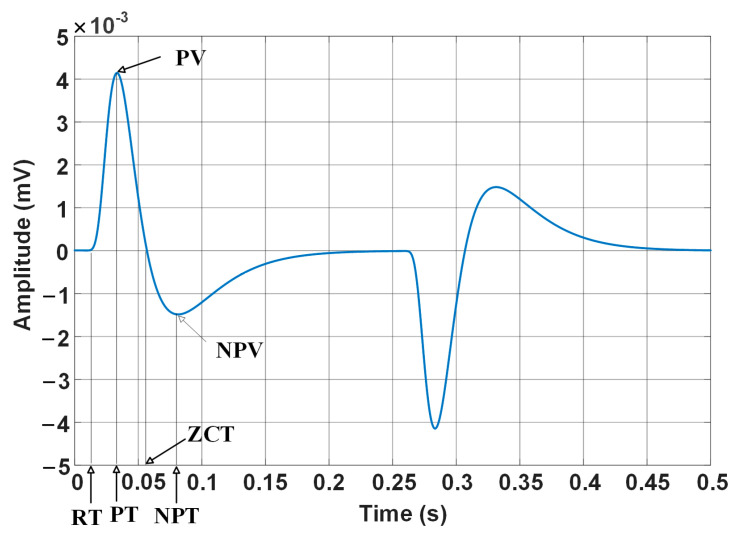
Plot of differential PECT signal and signal features.

Since the carbon steel is ferromagnetic while the stainless steel is non-ferromagnetic, the excitation magnetic field generated by the probe transmitter coil will be directed into the carbon steel base, meaning the inner liner is almost shielded from the magnetic flux. Thus, the outer carbon steel base tube has the dominant effect on the probe induction signal, while the inner stainless-steel liner has little effect on the induction signal. In order to highlight the signal from the liner, the base tube is used as the reference block, and the signal acquired on the reference block is subtracted from the acquired absolute PECT signal. Figure 2 shows the differential PECT signal. It can be seen that the signal has diverse features, including peak voltage (PV), peak time (PT), rise time (RT), zero-crossing time (ZCT), negative peak voltage (NPV), and negative peak time (NPT). Since the signal curve is oddly symmetric, the first half ranging from 0 to 0.25 s is analyzed.

### 3.1. Liner Wall Thinning

**Figure 3:**
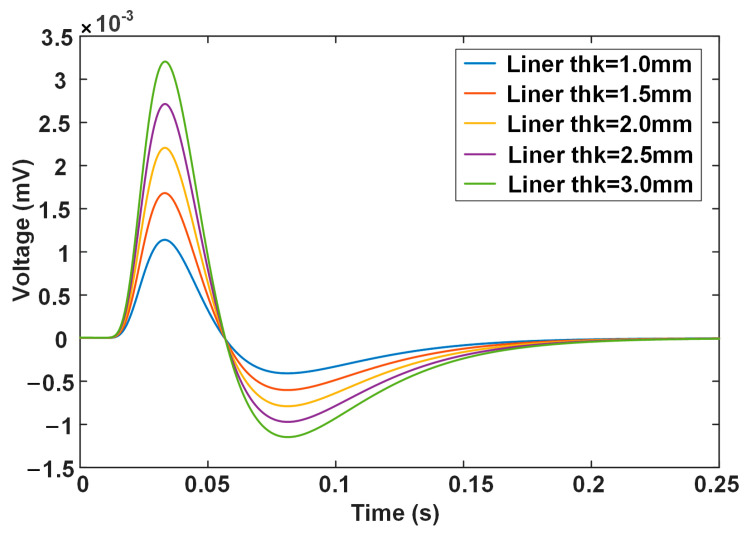
Calculated differential PECT signals for the different liner thicknesses.

Figure 3 shows the differential signals when the liner wall thickness is 1.0, 1.5, 2.0, 2.5, and 3.0 mm, respectively. The corresponding amounts of wall thinning are 3.0, 2.5, 2.0, 1.5, and 1.0 mm, respectively. The voltage becomes larger as the liner thickness increases, which reflects the fact that a thicker specimen produces a stronger reaction of eddy currents to the excitation field.

**Figure 4:**
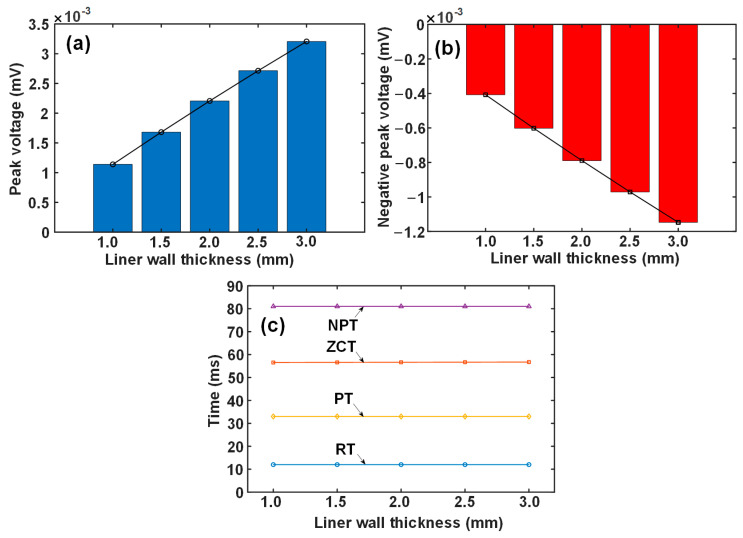
Variation in signal features with the liner wall thickness: (**a**) PV, (**b**) NPV, and (**c**) time-related features.

It is observed that the liner wall thickness has a monotonous relationship with the signal features, including PV and NPV, but the time-related features, including RT, ZCT, PT, and NPT, remain unchanged. Figure 4 plots the variation in the signal features with the liner wall thickness. It is clear that the PV and NPV values vary linearly with the liner wall thickness, which means that they can serve as features to characterize the liner wall thinning. However, PV shows a larger change rate (i.e., the slope of the line) with the liner wall thickness, which indicates a better sensitivity than NPV.

### 3.2. Interface Debonding

**Figure 5:**
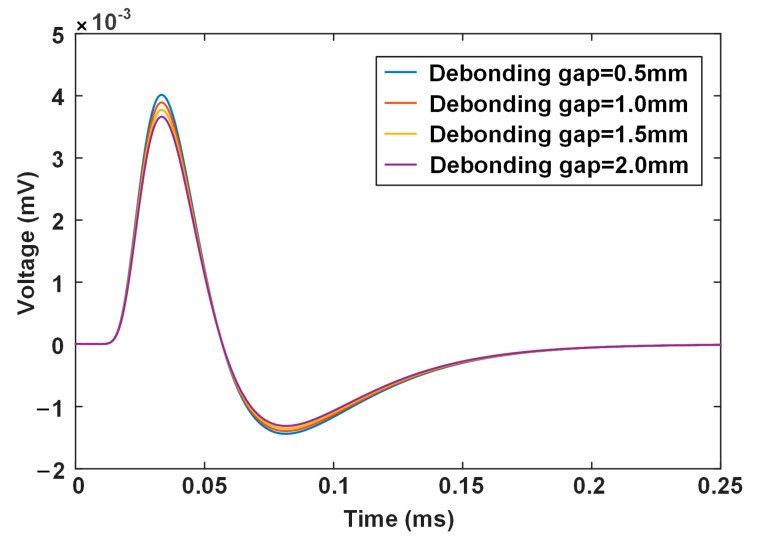
Calculated differential PECT signals for different debonding gaps.

The interface debonding can be simulated by setting an air gap between the base and the liner. Figure 5 depicts the calculated differential signals when the interface gap varies from 0.5 to 2.0 mm. In contrast to liner wall thinning, the debonding defect brings about a smaller response on the probe signal. The larger the gap is, the smaller the signal voltage is. Again, what can be perceptibly seen in terms of change is the amplitude-related features, PV and NPV.

**Figure 6:**
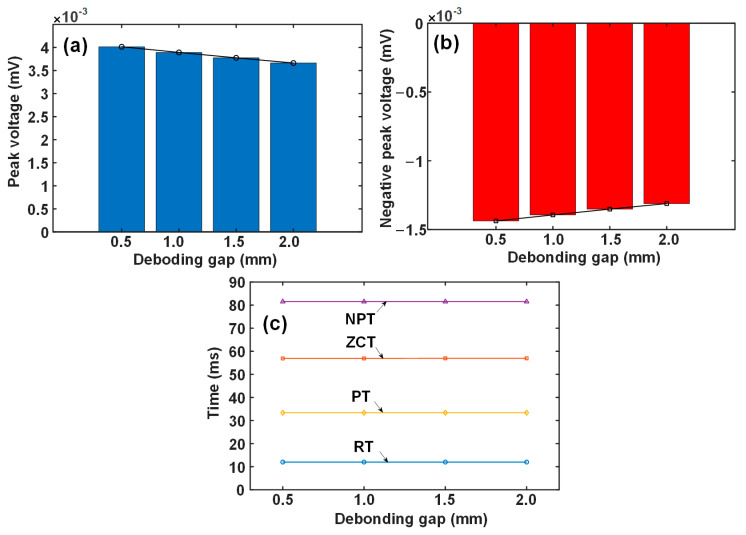
Variation in signal features with the debonding gap: (**a**) PV, (**b**) NPV, and (**c**) time-related features.

Figure 6 plots the variation in signal features against the debonding gap. The time-related features are again invariable when the debonding gap increases. The change in peak voltage is more evident than that of the negative peak voltage, but the amount is generally small. When the debonding gap increases from 0.5 to 2.0 mm, the decrease in peak voltage is less than 0.5 microvolts, which is a very weak variation and challenges the subsequent signal processing, including conditioning and data acquisition. The reason can also be attributed to the magnetic shunt by the carbon steel, rendering the response due to debonding hardly able to reach the probe placed outside.

## 4. Experimental Verification

**Figure 7:**
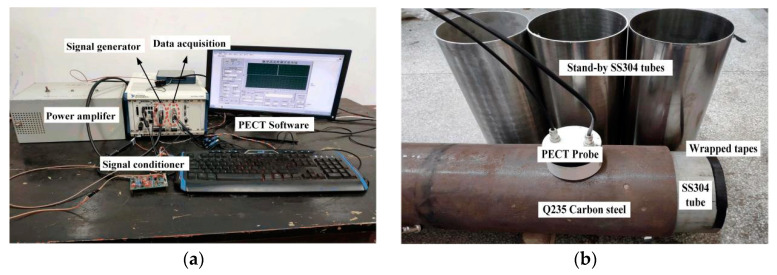
(**a**) PECT platform; (**b**) specimen.

Figure 7 shows photographs of the PECT platform and specimen. The platform mainly consists of three sections: signal generation, probe–specimen interaction, and data acquisition. A square-wave voltage signal with preset parameters is output by a signal generator card (PXIe-6368, National Instruments, Austin, TX, USA), then converted to a current signal and amplified by a custom-made power amplifier. The transmitter coil of the probe carries the amplified square-wave current and interacts with the specimen in the form of eddy currents. The secondary magnetic field generated by eddy currents is sensed by the receiver coil. This receiving voltage signal is preprocessed by a customized signal conditioner and then interfaced to a data acquisition card (DAC, PXIe-4497, National Instruments, Austin, TX, USA). The system is embedded with a software, which provides flexible functions, including real-time display, data storage for off-line analysis, and feature extraction. The probe has the same coil parameters as used in the simulation. 

The double-walled specimen is simulated by a combination of a Q235 carbon steel (equivalent to AISI1015) pipe (DN219 mm, thickness 10 mm, length 2 m) and SS304 tubes of different sizes. In the scenario of liner wall thinning, the inner SS304 tube has an outer diameter equal to the inner diameter of the carbon steel pipe and a varied wall thickness, while in the scenario of debonding, the inner SS304 tubes are of the same wall thickness, but the outer diameter is varied. For the latter case, the SS304 tube and Q235 carbon steel pipe are co-axially placed by padding nonconducting tapes into the gap between them.

During the experiment, the PECT signal can be easily disturbed by various factors, including probe vibration, tilting, and surrounding electromagnetic interference, which can contaminate the accuracy of the extraction of peak values. In order to increase the signal-to-noise ratio, the raw signals were averaged over 20 pulse cycles. However, the probe vibration is synchronized with the PECT pulses as it is generated by the pulses themselves. Each time the PECT probe emits a pulse, the probe vibrates due to the reaction force caused by the magnetostriction of the carbon steel base tube. Hence, it cannot be eliminated with averaging over multiple pulses [30]. In this context, the probe coils were fitted with a nylon case, and the probe case was pressed tightly on the base tube during the inspection. This allowed us to dampen the probe vibrations, effectively lowering the noise on the PECT signal.

**Figure 8:**
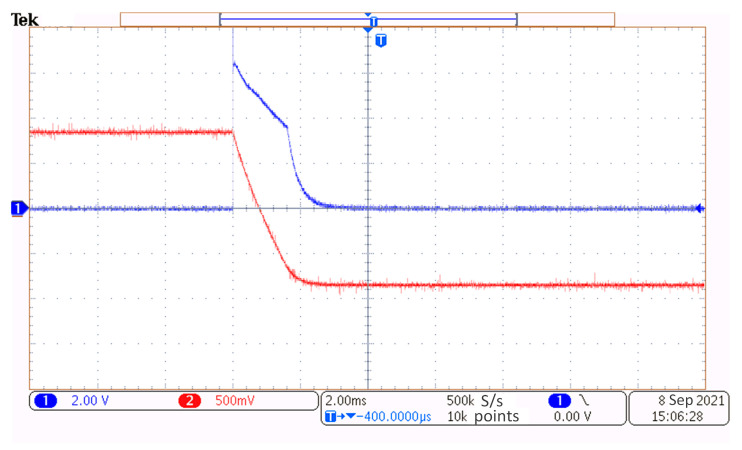
Screenshot of the oscilloscope graphing the excitation current and induced voltage signals.

Figure 8 shows a screenshot of the oscilloscope (MDO3012, Tektronix, Tokyo, Japan) monitoring the excitation current and the induced voltage in the PECT probe. Note that the displayed signals are raw signals with rough noises. The excitation current (marked in red color) is measured by a 0.25 ohms sampling resistor connected in series with the transmitter coil. It is observed that the current waveform is trailed to last about 2 ms at the edges due to the coil inductance. The receiver coil generates a voltage pulse (marked in blue) at the edge of the excitation current. It appears to be a typical ferromagnetic PECT signal, in which the response from the stainless-steel liner is too weak to be manifested.

As mentioned above, the responses caused by the liner wall thinning or debonding are very weak, commonly in microvolts. To unveil this rather weak signal, two techniques are applied. First, the signal from the base pipe (carbon steel) is acquired at the very beginning and used as the reference signal, and the subsequent signal from the bimetallic lined pipe is subtracted by the reference signal to generate a liner-only response signal. The second means is to utilize a customized signal conditioner with a gain of about 1000 and low-pass filtering of 1 kHz cut-off frequency to amplify the signal to the range of the DAC and further suppress the noise.

**Figure 9:**
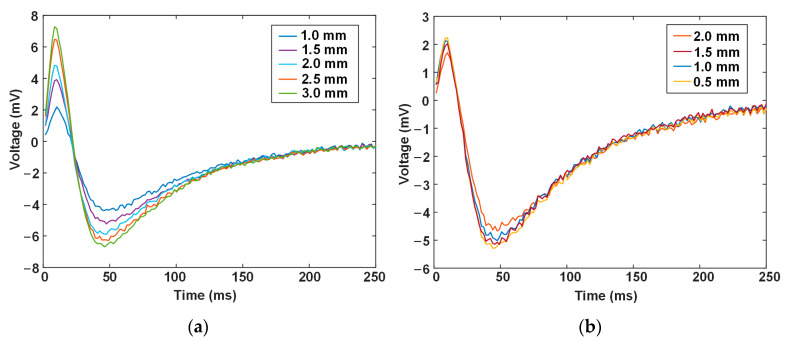
Experimental PECT signals for different amounts of (**a**) liner wall thickness and (**b**) debonding gap.

Figure 9 shows the experimental signals acquired from the cases of liner wall thinning and interface debonding. The characteristic of the signal waveform basically agrees with that made by analytical modeling: PV and NPV are revealed as the most noticeable features, and the signal variation is apparent with the liner wall thickness but subtle with the debonding gap. It is also noticed that the ratio of the positive peak to the negative peak is different from that in Figure 3 and Figure 5. The discrepancy might be attributed to the remanence effect of the carbon steel base tube. For the duration of the excitation pulse, the base tube will be magnetized; when the current switches off, the residual magnetic field in the base tube will introduce an extra magnetic flux in the probe coil, thus causing an offset to the voltage signal induced in the probe coil. In the simulation, the materials are considered to be linear, and, thus, the remanence effect is not included in the probe signal.

**Figure 10:**
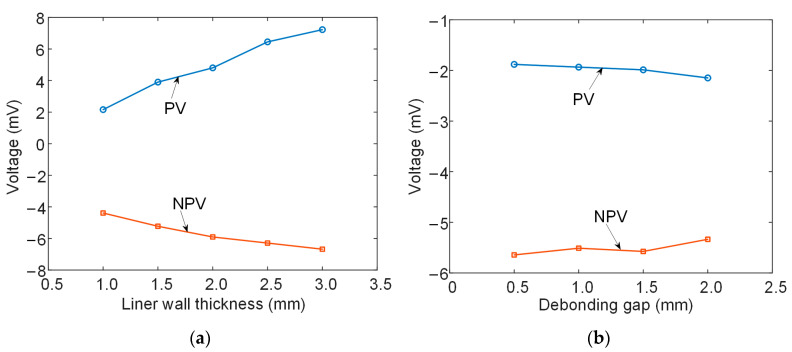
Variation in experimental signal PVs and NPVs with (**a**) liner wall thickness and (**b**) debonding gap.

Figure 10 depicts the extracted PVs and NPVs of the signals against the liner wall thickness and debonding gap. Bear in mind that the original experimental signals are rather weak (as predicted by the simulation) and, thus, are amplified 1000-times by the signal conditioner. For this reason, the amplified voltages are in the range of mV rather than 10^−3^ mV obtained from the simulation. Considering this condition, the monotonous variations and variation trends are in good accordance with the modeling results, with an exception for the NPV values acquired at debonding gaps of 1.0 and 1.5 mm. The reason for this exception could probably be related to the small change in the signal itself. In general, the PV value shows a more significant and measurable change for the two cases, thus validating the effectiveness of using the positive peak value of the PECT signals to evaluate the liner wall thinning and interface debonding in BLPs. 

## 5. Conclusions

This work briefly studied the use of the pulsed eddy current testing (PECT) technique in evaluating the inner defects of bimetallic lined pipes (BLPs) from outside the pipe. The two commonly occurring defects in BLPs, liner wall thinning and interface debonding, were successfully inspected. The layered pipe was simplified as a stratified conductive plate, and analytical PECT signals were used to predict the signal behaviors. It was found that the differential signal exhibited diverse signal features: the peak value, negative peak value, rise time, peak time, time to zero-crossing, and time to negative peak. The peak value was employed to characterize the defects because it showed a linear and relatively larger variation with the remaining liner thickness and debonding gap. Experiments were also performed to verify the analytical modeling, given that the double-walled specimen was simulated by assembling a Q235 carbon steel pipe as the casing and SS304 tube as the insert. The peak value of the PECT signal shows linear increasing and decreasing changes with the increase in the remaining liner wall thickness and debonding gap, respectively, while the negative peak value shows opposite changes. The peak value is superior to the negative peak value due to its larger sensitivity. The presented work demonstrates that PECT is a potential NDT method for practical applications in the evaluation of inner defects in BLP lines. 

In contrast with existing NDT strategies [11,12,13,14,15], the proposed technique has advantages as follows. Since the inspection is implemented from the outside of the pipe, the work conditions in the pipe have little effect on the signal responses of the external probe. Secondly, the probe can be quickly and easily placed onto the interrogated pipes without spraying a couplant and surface preparation. Thirdly, the deep penetration of pulsed eddy currents means that the technique is capable of simultaneously evaluating the defects in the base tube, interface, and liner.

The current work demonstrated the feasibility of using peak values of the PECT differential signal to detect the liner wall thinning and interface debonding, but, as inferred from Figure 10, it is still impossible to separate the detected deviation by using signs and the variation amount of the signal in the thinning of the liner as well as changes in the intertube gap. Meanwhile, the concurrent case of liner thinning and interface debonding should also be explored. With the ultimate goal of defect classification and quantitative evaluation, more comprehensive and multi-tier studies need to be carried out. 

For the other type of bimetallic composite pipes, namely hot-rolled clad pipes, their interfaces are metallurgically bonded and, therefore, less likely to introduce debonding defects. Whether the PECT signal exhibits similar variation in the amplitude- and time-related features when inspecting the liner wall thinning will be examined in the near future.

## Figures and Tables

**Table 1:** Electromagnetic properties of the specimen.

	CS Base	CRA Liner
Electrical conductivity σ (MS/m)	5	1.37
Relative magnetic permeability $\mu_{r}$	350	1

**Table 2:** Probe coil parameters.

	Transmitter Coil	Receiver Coil
Inner radius *r*_1_ (mm)	9.5	2.7
Outer radius *r*_2_ (mm)	19.5	5.4
Height *l*_2_ − *l*_1_ (mm)	35	6.3
Wire size (AWG)	20	40
No. of turns of wire	500	2000

## Data Availability

The data that support the findings of this study are available from the first author.
